# Endoplasmic Reticulum Stress in the Diabetic Kidney, the Good, the Bad and the Ugly

**DOI:** 10.3390/jcm4040715

**Published:** 2015-04-20

**Authors:** Robyn Cunard

**Affiliations:** 1 Research Service and Division of Nephrology-Hypertension, Veterans Affairs San Diego Healthcare System, Veterans Medical Research Foundation, San Diego, CA 92161, USA; 2 Department of Medicine, University of California San Diego, La Jolla, CA 92093, USA

**Keywords:** unfolded protein response, albuminuria, inflammation, mTOR, autophagy

## Abstract

Diabetic kidney disease is the leading worldwide cause of end stage kidney disease and a growing public health challenge. The diabetic kidney is exposed to many environmental stressors and each cell type has developed intricate signaling systems designed to restore optimal cellular function. The unfolded protein response (UPR) is a homeostatic pathway that regulates endoplasmic reticulum (ER) membrane structure and secretory function. Studies suggest that the UPR is activated in the diabetic kidney to restore normal ER function and viability. However, when the cell is continuously stressed in an environment that lies outside of its normal physiological range, then the UPR is known as the ER stress response. The UPR reduces protein synthesis, augments the ER folding capacity and downregulates mRNA expression of genes by multiple pathways. Aberrant activation of ER stress can also induce inflammation and cellular apoptosis, and modify signaling of protective processes such as autophagy and mTORC activation. The following review will discuss our current understanding of ER stress in the diabetic kidney and explore novel means of modulating ER stress and its interacting signaling cascades with the overall goal of identifying therapeutic strategies that will improve outcomes in diabetic nephropathy.

## 1. Introduction

Diabetic kidney disease (DKD) is the most common worldwide cause of chronic kidney disease and a growing public health challenge [1]. The development of diabetic nephropathy is influenced by a number of factors including genetic and environmental susceptibility, altered glomerular and tubular hemodynamics, epigenetic mechanisms and inflammation. Moreover, the micro- and macro-environment in the diabetic individual is greatly perturbed in DKD leading to the dysregulation of many homeostatic signaling pathways.

The endoplasmic reticulum (ER) is the central site for folding, post-translational modifications, and transport of secretory, luminal and membrane proteins. The ER is also involved in calcium storage and lipid biosynthesis. Over two decades ago investigators observed that the accumulation of misfolded proteins in the ER, induced the expression of glucose-regulated proteins (GRPs) including GRP78/heavy chain binding protein (BiP) [2,3]. Subsequent work revealed that increases in the load of unfolded proteins in the ER, activates a complex signaling pathway—the unfolded protein response (UPR). The UPR increases the folding capacity of the ER by upregulating the mRNA translation of ER chaperones and inhibiting the translation of most proteins. The coordinated activation of these pathways is designed to rapidly reduce the ER load. The UPR is a homeostatic pathway that regulates ER membrane structure and secretory protein processing capacity in a dynamic and coordinated manner [4,5]. When the cell becomes overwhelmed by misfolded proteins, then it is considered the ER stress response. However, it is unclear when cells cross the line between the UPR and ER stress; Rutkowski and Hedge have suggested that ER stress occurs when the ER functions in an environment that lies outside of its normal physiological range.

A number of factors activate the UPR including nutrient excess and deprivation, altered protein glycosylation, reducing agents, changes in ER calcium content, oxidative stress and TLR signaling. The diabetic milieu is associated with aberrant protein folding and activation of the UPR. Bacterial and viral infections and bacterial toxins activate the UPR by a variety of mechanisms (reviewed in [6]). Additionally, microRNAs (miRNAs) modulate the UPR and may play a role in the switch between the homeostatic UPR and ER stress [7,8,9]. However our understanding of microRNAs and their impact on the UPR remains rudimentary.

## 2. Mammalian UPR

In mammalian cells, there are three major arms of the UPR: (1) protein kinase RNA (PKR)-like ER kinase (PERK); (2) inositol requiring protein-1α (IRE1α) and (3) activating transcription factor-6 (ATF6) pathways. The PERK pathway rapidly attenuates protein translation, whereas the ATF6 and the IRE1α cascades transcriptionally upregulate ER chaperone genes that promote proper folding and ER-associated degradation (ERAD) of proteins, allowing the folding machinery of the ER to catch up with the backlog of unfolded proteins. These pathways are designed to relieve the accumulation of misfolded ER proteins, however when these pathways are overwhelmed by sustained ER stress, the UPR initiates pro-apoptotic pathways [10,11,12,13,14]. The UPR is also associated with adaptive processes such as IRE-dependent Decay (RIDD) and MicroRNA-dependent gene silencing [7,8].

GRP78/BiP is an ER chaperone protein that activates the UPR. In unstressed cells, GRP78/BiP binds to the ER luminal domains of the ER stress sensors: IRE1α, PERK and ATF-6 and maintains them in an inactivated state [15,16]. During ER stress, BiP preferentially binds to unfolded and misfolded proteins and dissociates from the transmembrane sensors, facilitating their activation. After BiP dissociation, it is not clear whether full activation of the UPR requires subsequent binding of unfolded proteins to the luminal domains of IRE1α, PERK and ATF-6 (reviewed in [17,18]).

## 3. PERK Pathway

PERK is a transmembrane protein with an ER luminal stress-sensing domain that binds GRP78/BiP, and a cytosolic kinase domain [15]. When ER stress is sensed, PERK multimerizes and phosphorylates eukaryotic translation initiation factor 2α (eIF2α) [19]. Phosphorylation of eIF2α initially suppresses the translation of 90% of cellular mRNAs by interfering with 5ʹ cap assembly [5,19,20]. However, a subset of genes including activating transcription factor-4 (ATF4) [19] and nephrin [21] are preferentially translated when eIF2α is phosphorylated (p-eIF2α). ATF4 a transcription factor, then binds to promoter/enhancer regions to transcriptionally upregulate expression of specific UPR target genes, which include C/EBP homologous protein (CHOP, C/EBPζ, DDIT, growth arrest and DNA damage (GADD) 153) [22,23,24], GADD34 [25], vascular endothelial growth factor (VEGF) A [26], TRB3 [27], osteocalcin, bone sialoprotein [28], receptor activator of NF-κB ligand (RANKL), E-selectin and genes important in amino acid metabolism [29,30]. Other stress-associated kinase signaling pathways converge downstream of p-eIF2α, thus p-eIF2α functions to induce an “integrated stress response” [17,29].

## 4. IRE1α/X Box Protein-1 (XBP-1) Pathway

The IRE1α/XBP-1 pathway is the most evolutionarily conserved of the ER stress pathways [31]. IRE1α is a membrane-bound serine/threonine kinase with endonuclease activity [32,33]. When ER stress is sensed GRP78/BiP dissociates from IRE1α [34] and IRE1α splices a 26 bp intron from XBP-1. Splicing of XBP-1 induces a translational frame-shift that generates a 54 kd highly active transcription factor, compared with a smaller poorly active unspliced XBP-1 (uXBP) [35,36]. XBP-1 induces the transcription of genes involved in ER maintenance, ER expansion and ER associated degradation (ERAD) [37,38]. Studies suggest that uXBP may negatively regulate the UPR by binding and excluding spliced XBP-1 (sXBP) from the nucleus [39]. However, uXBP is inherently unstable and rapidly degraded in cells [40]. IRE1α also activates apoptosis signal-regulating kinase (ASK1), c-Jun *N*-terminal kinase (JNK) and nuclear factor kappa-light-chain-enhancer of activated B cells (NF-κB, reviewed in [41]), which are involved in apoptotic, autophagy and inflammatory pathways [42,43,44,45,46].

XBP-1 is member of the basic leucine finger cAMP Response element (CREB)/ATF transcription factor family and it is ubiquitously expressed. Not surprisingly, in mice XBP-1 deficiency is an embryonically lethal mutation due to liver failure [47]. XBP-1 plays a role in hepatic [48], plasma cell [47,49,50], dendritic [51,52] and effector CD8+ T cell [53] development, however its function in the kidney is poorly understood. XBP-1 preserves cell survival during the UPR, however after prolonged stress the IRE1α/XBP-1 arm of the UPR is attenuated, sensitizing the cells to apoptosis mediated by the PERK/CHOP pathway [54,55]. XBP-1 regulates the biogenesis and expansion of the ER and Golgi, is a key modulator of secretory cell function [47,56] and regulates genes involved in redox homeostasis and oxidative stress responses including catalase [38,57]. XBP-1 regulates VEGFA expression and is directly recruited to the VEGFA promoter under ER stress conditions [58]. VEGFA rapidly activates all three ER stress sensors (IRE1α, PERK and ATF6) and promotes endothelial survival [59]. XBP-1 also regulates hepatic glucose metabolism and the hexosamine biosynthetic pathway [60,61,62]. In nematodes XBP-1 increases longevity [63] and recent work suggests that XBP-1 binds to Hypoxia-inducible Factor 1α (HIF1α) to promote the growth of triple negative breast cancer [64].

## 5. Regulated IRE1-Dependent Decay (RIDD)

A subset of ER-localized mRNAs encoding secreted and transmembrane ER proteins are cleaved directly by the endonuclease activity of IRE1α. The cleaved mRNAs are then rapidly degraded in a process called regulated IRE1-dependent decay (RIDD) [65,66,67]. This process may assist the PERK arm of the UPR to reduce the accumulation of misfolded proteins in the ER. RIDD activity may also induce the rapid clearance of microRNAs (miRNA-17, -34a, -96 and -125b) that repress translation of caspase-2, to enhance expression of this pro-apoptotic protein [68]. RIDD activity also activates the Nod-like receptor family, pyrin domain containing 3 (NLRP3) inflammasome and promotes inflammation and programmed cell death [69]. However, the relevance of RIDD activity in the kidney is unknown.

## 6. ATF6

ATF6 is the third ER stress sensor that is bound as an inactive precursor in the ER membrane. During ER stress ATF6 is transported to the Golgi and cleaved by site 1 protease (S1P) and site 2 protease (S2P). Cleavage of ATF6 releases its cytoplasmic bZIP domain [70], which translocates to the nucleus and activates the transcription of target genes which include GRP78/BiP, XBP-1, GRP94, oxygen-regulated protein 150 (ORP150), ER oxidoreductin β (ERO1β), p58^IPK^ and degradation in ER protein 3 (Derlin 3) [16,36,71,72,73,74,75,76]. Interestingly, the Golgi localized proteases S1P and S2P also catalyze the proteolytic activation of a group of transcription factors, the sterol regulatory binding proteins (SREBPs), linking the ER stress response with lipid and cholesterol synthesis [77,78]. However, it remains unclear whether the UPR and SREBP pathways function in an antagonistic or synergistic manner [17].

## 7. ER Stress in the Kidney

Investigating the ER stress response in the kidney provides a number of challenges. The kidney has many diverse cell types [79] and the UPR functions in a very cell-type and context-dependent manner modulating different downstream pathways to restore tissue homeostasis [6]. However, the UPR likely plays a more significant role in secretory cells such as podocytes, which secrete many factors including VEGF, cytokines, chemokines and factors that promote glomerular basement membrane integrity [80,81]. Many seminal UPR studies have been performed in undifferentiated murine embryonic fibroblasts (MEFs), which likely do not model function in fully differentiated renal cells. Another challenge is regarding the complexity of the UPR with three known signaling pathways each with its own dynamic expression, oscillations and function [5,81]. Questions also remain regarding whether investigators should measure mRNA or protein expression of ER-relevant molecules when investigating the UPR, though it seems prudent to investigate both mRNA and protein expression. Finally, certain paradigms in the field have emerged, that are not consistent. An example is that expression of the transcription factor CHOP inexorably leads to cell death. However CHOP does not promote apoptosis in all cell types [82] and CHOP’s biological effects may be dependent on CHOP’s binding partner [83] or on the phosphorylation status eIF2α [84].

## 8. Renal Cell Systems

In murine podocytes hyperglycemia, advanced glycation end products (AGE) and free fatty acids (FFA) induce ER stress and apoptosis, and this can be inhibited by exogenous ER chaperones [82,85,86,87]. AGE increase intracellular calcium concentrations by releasing ER stores, and by increasing calcium influx of extracellular calcium [85]. FFAs induce GRP78/BiP and CHOP expression. CHOP can mediate apoptosis (depending on the phosphorylation status of eIF2α [84]) or transcriptionally activate downstream ER stress-associated genes including TRB3 [82,87]. In human proximal tubular cells (HK-2) palmitic acid (a FFA) induces ER stress and this can be blocked with a cannabinoid receptor antagonist [88]. In a human renal tubular cell line (HKC), high glucose conditions increase splicing of XBP-1 and transfection of spliced XBP-1 increases expression of fatty acid synthase and acetyl-CoA carboxlase to promote lipid synthesis [89]. In contrast, in mesangial cells, high glucose conditions reduce spliced XBP-1 expression. Transfection of adenoviral XBP-1 reverses high glucose-induced reactive oxygen species (ROS) production and extracellular matrix (ECM) expression. Whereas, knockdown of intrinsic XBP-1 increases ROS and ECM [90], supporting the renoprotective effects of XBP-1. These contrasting effects of high glucose conditions on XBP-1 splicing further illustrate the variable nature of ER stress responses and their strict dependence on cell type and cellular conditions. In human renal tubular cells (HK-2), ER stress increases oxidative stress and reduces anti-oxidant enzymes by reducing micro-RNA (miR)-205 expression [91]. Likewise in rat renal tubular cells (NRK-52E) Dehydroxymethylepoxyquinomicin (DHMEQ, an NF-κB inhibitor), increases ROS, which rapidly induce all three arms of the UPR [92]. Thus ROS activate the UPR [82,92] and depending on the cellular context and conditions the UPR can either negatively [90] or positively regulate generation of ROS [91].

## 9. ER Stress in the Diabetic Rodent Kidney

Mice with constitutive mutations in ER stress proteins develop diabetes, defects in glucose handling [93,94,95,96] and ER Stress induces β-cell failure [97,98,99]. Thus studies investigating the functional relevance of these proteins in DKD will need to employ conditional knockout or transgenic expression of key ER stress proteins (PERK, IRE1α, XBP-1, eIF2α *etc.*). Akita mice (Ins2^+/C96y^), used in studies of Type 1 DKD, have a missense mutation in the insulin gene, which causes accumulation of misfolded insulin, activation of ER Stress and subsequent pancreatic β cell failure [100,101,102,103]. Mice with a heterozygous constitutive knock-in of a mutant GRP78/BiP have evidence of ER Stress in the kidney, associated with age-related renal tubular atrophy, interstitial fibrosis and glomerulosclerosis [104].

A number of groups, including ours have documented activation of the ER stress response in the diabetic kidney [82]. Liu and colleagues were the first to evaluate the ER stress response in a mammalian model of diabetes. In STZ-treated rats (65 mg/kg STZ IP once), they demonstrated increased expression of GRP78/BiP in glomerular and tubular cells and enhanced kidney cell apoptosis, CHOP, JNK and caspase-12 expression [105]. In older STZ-treated mice (50 μg/g STZ for 5–8 injections), GRP78/BiP, CHOP, phosphorylated-PERK and p-eIF2α were increased in 22 month-old diabetic mice, compared with 9 month-old diabetic and non-diabetic mice. Diabetic CHOP knockout mice also had less proteinuria [106]. These studies consistently observed activation of ER stress in the diabetic kidney, but they did not elucidate whether it was protective or destructive. TRB3 is an ER stress-associated protein that is upregulated by free fatty acids and ROS through the PERK/CHOP UPR pathway [82]. We recently demonstrated that constitutive knockout of TRB3 worsens albuminuria, cytokine and chemokine expression in murine Type 1 diabetic kidney disease [107], supporting the protective effects of ER stress. Further studies in mice with transgenic and conditional knockouts of key ER stress-associated molecules will likely clarify the roles that these complex pathways play in the diabetic kidney.

Spliced XBP-1 is reduced in the renal cortices 8 weeks after STZ treatment in rats (65 mg/kg once) [90] and the authors hypothesize that this increases ROS (through nicotinamide adenine dinucleotide phosphate (NADPH) oxidase) and ECM. Our group has also observed lower XBP-1 mRNA expression in diabetic mouse kidneys (STZ and *db/db*, unpublished observations), though Chen observed higher spliced XBP-1 protein in renal cortices of *db/db* mice [108]. Interestingly, in hippocampuses of *db/db* mice microarray screening showed lower spliced and unspliced XBP-1 [109]. Variable findings of XBP-1 in the kidneys could be related to poor specificity of commercially available antibodies for XBP-1, highlighting the importance of verifying XBP-1 expression by real-time PCR. It is not clear why XBP-1 expression is downregulated in the diabetic kidney. However ER stress has distinct temporal patterns [107]. XBP-1 preserves cell survival during the UPR, and prolonged stress attenuates the IRE1α/XBP-1 arm of the UPR, sensitizing cells to apoptosis [54,55]. Thus strategies that augment the IRE1α/XBP-1 pathway may slow or prevent the progression of DKD.

## 10. ER Stress in Human Diabetic Kidney Disease

Few studies have investigated the ER stress response in the human diabetic kidney. Lindenmeyer and colleagues demonstrated that mRNA expression of GRP78/BiP, ORP150/HYOU1, S1P (MBTPS1), calnexin and XBP-1 increase in the kidneys of patients with established diabetes, compared with mild diabetes [110]. In human kidney transplant biopsies performed before implantation, GRP78/BiP was co-expressed with the inflammatory transcription factor NF-κB p65/RelA, suggesting that ER stress (induced by cold ischemia) activates tubular inflammation in human renal allografts [111]. Further studies investigating the impact and role of ER stress in human diabetic kidneys are needed. Wolcott-Rallison disease is caused by autosomal recessive mutations in PERK [112]. In this inherited defect of the UPR, children develop skeletal abnormalities and infantile diabetes. There are reports of renal insufficiency including proteinuria, which could indicate podocyte or tubular epithelial cell dysfunction or prerenal azotemia [113,114].

## 11. The UPR Interacts with a Number of Signaling Cascades

### 11.1. Inflammation and Immunity

Studies have demonstrated close links between ER stress activation and inflammation [115,116]. Moreover, enhanced inflammation plays a pathophysiological role in the diabetic kidney [117]. NF-κB drives the transcription of a number of cytokines and inflammatory molecules and all three arms of the UPR modulate NF-κB activity [118,119,120,121,122]. However the IRE1α/XBP-1 pathway seems to exert the most effect on inflammation and immunity to pathogens, by regulating the development of many inflammatory cell types [49,50,51,52,53] (reviewed in [6]). The IRE1α/XBP-1 pathway promotes immune tolerance in the gut [123], and may “protect against ER toxicity caused by innate inflammatory pathways” [124]. ER stress generates ROS, which activate cytokines/chemokines to drive many inflammatory responses. In human renal cortical tubular cells glucose deprivation activates the UPR, which promotes the transcription of interleukin (IL)-6, IL-8, tumor necrosis factor (TNF)-α, regulated on activation, normal T cell expressed and secreted (RANTES) and monocyte chemokine protein (MCP)-1 via NF-κB in an IRE1α-dependent manner [111]. In type 2 diabetic (*db/db*) mice ER stress triggers the expression of MCP-1 by XBP-1-mediated induction of SET7/9 (a histone lysine methyltransferase), which increases histone 3 lysine 4 methylation of MCP-1 promoters in renal cortices of diabetic kidneys [108].

The inflammasome is an oligomer of proteins that activate pro-apoptotic caspases and inflammatory cytokines, and it plays a key role in the innate immune response [125]. During ER stress the IRE1α and PERK pathways induce thioredoxin-interacting protein (TXNIP), to activate the NLRP3 inflammasome [69,126]. In human biopsies of proteinuric renal disease (IgA nephropathy, minimal change disease, membranous nephropathy, and DKD), inflammasome-related proteins such as caspase 1, IL-1β and IL-18 were expressed in the distal and proximal tubules and expression positively correlated with the degree of proteinuria [127]. In a tubular cellular model (NRK-52E), bovine serum albumin (BSA) induces expression of inflammatory cytokines, NLRP3, GRP78/BiP and phosphorylation of eIF2α. Indeed, use of a chemical chaperone to reduce ER stress attenuates inflammasome activation induced by albuminuria in a murine model of STZ-induced diabetic nephropathy, suggesting that ER stress also activates inflammation and kidney injury [127].

As previously discussed the transcription factor CHOP is classically considered to induce cellular apoptosis. However CHOP may inhibit inflammatory responses in the kidney, as mice deficient in CHOP expression develop more severe septic acute kidney injury (AKI) [128]. In contrast, CHOP’s function in chronic kidney disease is not completely understood [106]. Interestingly in human diabetic kidneys CHOP expression was not higher [110], though its expression is consistently augmented in murine models of diabetic kidney disease [82,105,106]. Future studies of ER stress in diabetic humanized rodent models may clarify some of these discrepant findings [129].

Toll-like receptors (TLRs) signal the existence of pathogens and activate the UPR to optimize ER function to facilitate high levels of secretory protein expression [124,130,131]. Indeed fungi and other organisms have used the UPR to provide virulence factors that support their survival in hostile environments [132]. Damage-associated molecular patterns (DAMP) are proteins released outside of the cell or expressed on the plasma membrane in stressed or damaged cells [133,134]. ER stress and ROS help traffic DAMPS to the plasma membrane, which recruit innate inflammatory cells to mediate immunogenic cell death. [135,136]. The relevance or existence of DAMP expression in the diabetic kidney has not been established, but it is tempting to speculate that like TLR’s, DAMPS may play a pathogenic role in DKD [137].

### 11.2. Mammalian Target of Rapamycin (mTOR)

mTOR is a conserved serine/threonine kinase modulated by growth factors and cellular energy status, and it is a constituent of mTOR complex 1 (mTORC1) and mTORC2. MTORC1 regulates growth, autophagy, survival and metabolism, whereas the role of mTORC2 is incompletely understood. mTORC1 activation in podocytes promotes the development of diabetic nephropathy [138,139] and it is associated with ER stress. The impact of mTORC2 activation in diabetic kidney disease remains unknown. However our group has shown that TRB3, an ER stress-associated protein binds to mTOR and the rapamycin-insensitive companion of mTOR (RICTOR), a protein specific to mTORC2, and inhibits inflammatory cytokine expression [107]. Absence of TRB3 also worsens albuminuria, cytokine and chemokine expression in DKD, another example of the salubrious effects of ER stress [107]. The interactions between ER stress and mTOR pathways is complex and has recently been carefully reviewed [140].

### 11.3. Autophagy

Macroautophagy (referred to as autophagy) is a cellular pathway that preserves homeostasis by degrading long-lived proteins and dysfunctional organelles [141,142]. Autophagy exerts both cytoprotective and cytocidal effects, and dysregulation of autophagy contributes to podocyte dysfunction in diabetic nephropathy [143]. For almost a decade investigators have identified links between ER stress and autophagy. Autophagy may be activated during ER stress to supplement ERAD [46,144,145]. Autophagy can originate from the ER membrane and be triggered by ER stress [44,146,147,148,149,150,151,152]. Interestingly in neurons, knockdown of XBP-1 activates autophagy [153], though in neuroglioma cells XBP-1 activates autophagy [154], again highlighting the cell specific effects of ER stress.

The link between ER stress and autophagy in renal pathophysiology was first described in renal tubular cells [155,156]. Podocytes have high levels of autophagy [157] and in cultured podocytes an ER stress inducer tunicamycin (TM), enhances microtubule-associated protein light chain 3 (LC3, a key autophagy protein) [158]. Hartleben and colleagues elegantly demonstrated that podocyte-specific deletion of autophagy-related 5 (Atg5) leads to glomerulopathy in aging mice and this was associated with ER stress, podocyte loss, proteinuria and glomerulosclerosis [143]. mTOR closely regulates autophagy, further supporting the tight interconnections among ER stress, mTOR and autophagy pathways [159,160].

## 12. Modulation of ER Stress

A number of therapeutic strategies designed to modulate ER stress have been employed in kidney diseases. Preconditioning with low doses of ER stress inducers TM and thapsigargin (TG) is protective in mesangioproliferative glomerulonephritis, Heymann’s nephritis and ischemia-reperfusion [161,162,163]. Preconditioning has not been studied in diabetic kidney disease, perhaps due to its chronic nature. However, in a model of diabetic retinopathy, the salutary effect of ER stress preconditioning in retinal endothelial cells is dependent on XBP-1 expression [164].

ER chaperones have been used therapeutically to promote protein folding and to reduce protein aggregation in the ER [165,166,167]. Tauroursodeoxycholic acid (taurine conjugate form of ursodeoxycholic acid, TUDCA) is a chemical chaperone that has been used in traditional Chinese medicine for a number of indications [168]. In murine podocytes TUDCA reduces AGE-induced expression of GRP78/BiP and podocyte apoptosis [85]. TUDCA restores defective autophagy and attenuates albuminuria and histopathological changes in diabetic mice, though these changes were associated with mild improvements in glucoses [169]. TUDCA also reduces inflammasome activation (suppression of caspase-1 activation, IL-1β and IL-18 maturation) in a murine model of DKD [127]. In a clinical study 4 weeks of TUDCA improves hepatic and muscle insulin sensitivity and signaling but did not change markers of ER stress in muscle or adipose tissue [170]. Renal effects were not studied.

In a rat model of diabetes 4-phenyl butyric acid (4-PBA, 1 mg/kg), an ER chaperone, attenuates manifestations of diabetic nephropathy including markers of renal oxidative stress such as NADPH oxidase activity, however the investigators only assessed one marker of ER stress (IRE1α) and 4-PBA treatment was associated with improved glycemic control and markers of renal function (BUN and creatinine) were unchanged [171]. In STZ-treated rats 4-PBA improves renal hypertrophy, hyperglycemia, urinary protein excretion and mesangial matrix expansion, however these changes were not associated with improvements in serum creatinine [172]. Nephrin is a large podocyte transmembrane protein that plays a key role in slit diaphragm integrity. Nephrin mutations, which cause congenital nephrosis can trigger the UPR, and 4-PBA facilitates plasma membrane expression of some nephrin mutants [173]. Indeed hyperactivation of mTORC1 (in diabetic nephropathy) is associated with ER stress and mislocalization of nephrin in podocytes. 4-PBA treatment of these mice significantly reduces GRP78/BiP expression and prevents podocyte loss, but does not normalize nephrin membrane localization or proteinuria [138]. In renal tubular epithelial cells 4-PBA decreases expression of GRP78/BIP, receptors for AGE (RAGE) and reduces premature senescence in cells pretreated with AGEs [174]. These studies demonstrate that ER chaperones modulate DKD, but it is unclear if their positive effects are related to improvements in glycemic control or direct modulation of ER stress in the kidney. However, they are encouraging and supportive of future human clinical studies in diabetic nephropathy.

Glucagon-like peptide-1 (Glp-1) is a metabolic hormone (incretin) secreted by intestinal cells. Liraglutide is a Glp-1 agonist used for the treatment of Type 2 diabetes and obesity. Liraglutide reduces ER stress in diabetic models of cardiomyopathy and pancreatic β-cell loss [175,176,177]. Additionally liraglutide is protective in diabetic kidney disease, though it is unclear if it modulates the UPR in the kidney [178,179]. Erlotinib, an epidermal growth factor receptor inhibitor slows the progression of murine diabetic nephropathy and this is associated with lower tubular and glomerular CHOP and lower glomerular GRP78/BiP and PERK expression. The downregulation in ER stress markers was associated with increased expression of key autophagy associated proteins including Atg12, beclin and LC3-II in renal glomeruli and tubules [180].

The XBP-1 arm of the ER stress response is generally cytoprotective, thus generalized downregulation of ER stress may not positively affect the progression of DKD. Strategies that augment XBP-1 activity may improve glycemic control and microvascular complications of diabetes [60]. A number of groups have synthesized small molecules that block IRE-1α activity and splicing of XBP-1 [181,182,183,184,185] for use in multiple myeloma and chronic lymphocytic leukemia, however their effect on diabetic nephropathy is unpredictable. Rapamycin (an mTOR inhibitor) may also selectively inhibit the IRE1α pathway [186] and rapamycin has been successfully used in rodent models of diabetic kidney disease [187,188,189,190,191]. However, rapamycin’s impact on the UPR has not been thoroughly evaluated, and its known effects of new-onset diabetes after transplantation (NODAT) and proteinuria may preclude its use in diabetics [192].

BiP inducer X (1-(3,4-dihydroxyohenyl)-2-thiocyanate-ethanone, BIX) is a small molecule GRP78/BiP inducer that also increases GRP94, calreticulin, and CHOP [193]. BIX activates the UPR selectively through ATF6, up-regulates renal GRP78/BiP expression and ameliorates renal ischemia-reperfusion injury [194]. Salubrinal, an inhibitor of eIF2α phosphatases, was discovered in a screen for drugs that prevent ER stress-induced apoptosis. Phosphorylation of eIF2α reduces protein synthesis and salubrinal protects cells from ER stress by maintaining high levels of phosphorylated eIF2α [195]. In podocytes salubrinal restores defective autophagy and podocin expression [169], though in cisplatin-induced nephrotoxicity, salubrinal enhances oxidative stress and renal injury [196]. Although BIX and salubrinal may provide benefit, their effects in DKD are unknown. Similar approaches to limit protein synthesis in the kidney may be theoretically beneficial in DKD [84]. RIDD activation reduces the toxicity of acetaminophen overdose by degrading key hepatic cytochrome enzymes which covert acetaminophen to its toxic metabolites [197]. The relevance of RIDD activity in the kidney is still not clear, but augmentation of RIDD activity may provide a novel therapeutic approach.

Angiotensin converting enzyme inhibitors (ACE I) and angiotensin receptor blockers (ARB) are currently used to slow the progression of DKD. In murine STZ-induced diabetes high dose irbesartan (an ARB) reduces ER stress and apoptosis in tubules [198]. In rat STZ-induced diabetes perindopril (an ACE I) reduces markers of ER stress and apoptosis in the tubulo-interstitium [199]. In contrast in rodent diabetic kidney disease low dose irbesartan reduces albuminuria and inflammation, but does not reduce ER stress (calnexin and GRP78/BiP staining) [200]. Valsartan (an ARB) and aliskiren (direct renin inhibitor) also reduce CHOP and XBP-1 expression in rodent diabetic kidneys [201]. Thus the beneficial effects of renin-angiotensin system blockade, may be in part related to modulation of ER stress.

Febuxostat is a non-purine inhibitor of xanthine oxidase and is used therapeutically for gout and hyperuricemia. In a rat ischemia-reperfusion model febuxostat improves renal function, inflammation and apoptosis by inhibiting oxidative and ER stress [202]. In rat STZ-induced diabetes febuxostat reduces albuminuria, glomerular macrophage infiltration and inflammation [203], though its effects on ER stress have not been reported. Recently Wang and colleagues demonstrated in male obese Zucker rats that low dose acetaminophen reduces ER-stress signaling, tubular and glomerular apoptosis and albuminuria [204]. Thus, some currently approved and widely-used medications such as ACE I, ARBs, febuxostat, liraglutide, rapamycin and low dose acetaminophen may modulate the UPR and attenuate the progression of DKD. However these agents may reduce renal injury and indirectly ER stress, thus further studies are indicated to evaluate their precise effects on ER stress pathways in the diabetic kidney.

## 13. Problems with UPR/ER Stress Studies in the Kidney

Many studies are based on the assumption that high levels of ER stress-related molecules denote active ER stress. However Rutkowski and Hedge have postulated that changes in ER stress-associated molecules may represent homeostatic fluctuations in the ER in response to rapidly changing environmental conditions [4]. Moreover, some studies investigate only one or two ER stress-related molecules and assume that the UPR is activated. In these studies more thorough investigative methods are indicated [205]. Our understanding of the UPR is also hindered by the difficulty in blocking or activating a single arm of the UPR, as the pathways are highly interconnected among themselves and with other downstream signaling cascades. It is also clear that ER stress is neither good nor bad for the kidney; one must be careful not to correlate downregulation of ER stress (by one or two ER stress markers) as beneficial, especially given the cyto-protective effects of XBP-1.

## 14. Future Directions and Conclusions

The UPR is very dynamic with a wide range of inputs and outputs that enable the cell to respond to a number of diverse stimuli and cellular conditions. ER stress is activated in the diabetic kidney and the UPR restores normal organ function in aberrant physiological conditions. However, chronic activation of these pathways likely contributes to chronic renal injury, inflammation and the progression of chronic kidney disease. Many questions remain regarding which renal cells are most affected by ER stress and whether activation of one pathway constitutes the ER stress response, or must all three arms of the UPR be activated? Moreover, few studies evaluate the temporal patterns of ER stress activation and it is likely that timing will have a profound effect on outcomes. The development of specific ER stress modulators that modify individual arms of the UPR will provide therapeutic strategies to treat the development and progression of DKD. Additionally, the ability to carefully amplify or reduce UPR activation may also be efficacious. Furthermore, focused studies investigating ER stress in humans or humanized rodent models will further our understanding of the UPR in the diabetic kidney.
